# Cultural adaptation of the mental health first aid guidelines for assisting a person at risk of suicide in Brazil: a Delphi expert consensus study

**DOI:** 10.1186/s12888-022-04042-7

**Published:** 2022-06-13

**Authors:** Simone Scotti Requena, Amanda Vidotto Cerqueira, Thais Alves Assumpção, Carlos Henrique Mesquita Peres, Alexandre Andrade Loch, Nicola J. Reavley

**Affiliations:** 1 Centre for Mental Health, Melbourne School of Population and Global Health, The University of Melbourne, Melbourne, Victoria 3010 Australia; 2 Laboratorio de Neurociencias (LIM 27), Instituto de Psiquiatria, Hospital das Clinicas HCFMUSP, Faculdade de Medicina, Universidade de São Paulo, São Paulo, Brazil; 3 Instituto Nacional de Biomarcadores em Neuropsiquiatria, Conselho Nacional de Desenvolvimento Cientifico e Tecnologico, São Paulo, Brazil

**Keywords:** Suicide, Mental health first aid, Cultural adaptation, Delphi study, Brazil

## Abstract

**Background:**

Suicide is a major public health concern in Brazil, with nearly 115,000 Brazilians dying by suicide in 2010–2019. As support for individuals at risk of suicide may come from the community, particularly family and friends, it is fundamental that evidence-based programs or resources to improve such support are in place when needed. This study aimed to culturally adapt the mental health first aid guidelines for assisting a person at risk of suicide used in English-speaking countries for Brazil.

**Methods:**

A Delphi expert consensus study was conducted among a diverse range of Brazilian health professionals and individuals with lived experience of suicide (*n* = 60). A total of 161 items from the mental health first aid questionnaire used in English-speaking countries were translated and used in the Brazilian questionnaire. Participants were asked to rate the appropriateness of those items to the Brazilian culture and to recommend any new items when appropriate.

**Results:**

Data were collected over two survey rounds. Consensus was achieved on 145 items. While 123 out of 161 items were adopted from the English guidelines, 22 new endorsed items were created from the expert panel comments.

**Conclusions:**

Even though there were similarities among the Brazilian and English-language guidelines, the adapted guidelines incorporated actions that were specific to the Brazilian culture, such as new items emphasising the role of family and friends. Further research is warranted on dissemination and uptake of the guidelines in Brazil as well as research into incorporation of the guidelines into Mental Health First Aid (MHFA) training for Brazil.

**Supplementary Information:**

The online version contains supplementary material available at 10.1186/s12888-022-04042-7.

## Background

Suicide is a serious global health issue. The World Health Organization estimates that over 700,000 cases of suicide occur every year worldwide [1]. In 2019, more than one in 100,000 deaths were due to suicide, [2] and it is the 17th leading cause of death across all age groups and the fourth among young people (15–19 years old) [3]. More than three-quarters of all suicides occur in low- and middle-income countries (LMICs) [2]. While suicide rates have decreased worldwide, with a 36% decrease between 2000 and 2019, this was not the trend in Latin America [2].

Even with suicide deaths being underreported and misclassified [4, 5], in Brazil, it is estimated that nearly 115,000 people died by suicide in 2010–2019, with a gradual increase (5.2 to 6.7 per 100,000) in suicide deaths over that period [6]. In 2019, young Brazilians (15–19 years) had the highest increase in suicide deaths (an 81% increase), with men being almost four times more likely to die by suicide compared to women (10.7 vs 2.9 per 100,000 individuals) [6].

Ecological studies conducted in Brazil showed that higher income inequality and higher socioeconomic status (such as higher levels of education) were related to a higher risk of suicide [7–9]. Another study conducted in the most populated city of Brazil (Sao Paulo) found other risk factors for suicide, such as relationship status (being single), migration status (being a migrant), and religious choice (being a Catholic) [8].

Evidence suggests that Latin America has culturally-specific dimensions that need to be considered to understand suicide in the region, including *familism*, *machismo* [10, 11], *compadrazgo*, and *dignidad y respeto* [11, 12].

It is argued that these cultural aspects may be shaping beliefs and behaviours related to suicide in the Latino culture [10]. For instance, *familism* advocates for a welfare system that places responsibility on the family members for financial and emotional support instead of the government, whereas *machismo* refers to a patriarchal society, where the man is responsible for providing for his family, and the woman is the head of the family unit [10]. *Compadrazgo* involves a “friendliness” that values warm and close friendships, which may be strengthened by exchanging favours, while *dignidad y respeto* promotes empathy and connection in relationships [11, 12]. These relationships are hierarchically formed (“hierarchy of deference”) with elders and parents higher than younger people [11].

Other key factors relevant to understanding suicidality in Latin American countries include the limited resources of the health system and the stigma associated with seeking help for suicide and mental health problems [10]. This may include stigmatizing attitudes towards people with mental health problems by primary healthcare professionals [13–15].

Fakuda et al. (2016) conducted a study, with more than 1000 children and adolescents (8–21 years old), in several states of Brazil that explored barriers to seeking help for mental health services. It was found that fear of stigmatization was the major barrier to seeking help for mental health treatment.  Prejudice against individuals with mental disorders is a major barrier to accessing health care services [16]. A 2018 systematic review of pathways to mental health care in Brazil reported family as an important factor in identification, the decision to seek help, and the choice of the initial source of help for mental health issues [17]. Evidence suggests that community support, including from family and friends, can be a protective factor against suicide [18, 19].

In recognition of this, the Mental Health First Aid (MHFA) course was developed to train people in the general population on how to help a person who is developing a mental health condition or is in a mental health crisis (e.g., at risk of suicide) [20]. MHFA training is based on the First Aid model and incorporates an action plan that can be implemented until professional support is obtained or the crisis ends [20]. The MHFA program began in Australia in 2000 and has spread to over 25 countries [21]. The course is based on mental health first aid guidelines that have been developed through Delphi expert consensus studies with mental health professionals and people with lived experience [22–25]. The Delphi expert consensus is a method of incorporating practice-based evidence into evidence-based practice in a systematic manner when experimental and epidemiological methods are not feasible [26]. However, most of the participants in these Delphi studies have been from high-income settings, and the cultural appropriateness for LMICs is unclear. In an effort to ensure that the guidelines are appropriate, they have been culturally adapted for China [27], Sri Lanka [28], India [29], Philippines [30], and collaborations in Brazil, Chile, and Argentina have been formed [21].

This study aimed to culturally adapt the mental health first aid guidelines for assisting a person at risk of suicide used in English-speaking countries for Brazil.

## Methods

### The Delphi method

A two-round Delphi method was used in this study. The Delphi method transforms the experts’ opinions in a field into group consensus [26, 31, 32]. Our expert panel consisted of health professionals and people with lived experience of suicide. The panel rated whether statements from the English-language guidelines were appropriate to be included in the Brazilian guidelines and were also asked to suggest new items when appropriate.

This study occurred in four stages: (1) questionnaire development for the round 1 survey; (2) panel identification and recruitment; (3) data collection and analysis; (4) guidelines development.

### Questionnaire development for the round 1 survey

The mental health first aid guidelines for assisting a person at risk of suicide used in English-speaking countries [33] were translated into the Portuguese language by one senior psychiatrist and four medical students. During translation, to improve readability and fluency in Portuguese, minor changes were made to some statements, such as: “*The first aider should not assume that the person will get better without help*” was translated into “*O(a) socorrista não deve supor que a pessoa irá melhorar sem ajuda*”, and “*The first aider should ask the suicidal person if they have a plan for suicide*” was translated into *“O(a) socorrista deve perguntar à pessoa suicida se ela tem um plano para o suicídio*”.

As per the English-language guidelines, the round 1 survey included 161 items divided into eight sections (an additional Table 1 shows this more in detail [see Additional file 1]).

**Table 1:** Characteristics of participants (*n* = 60)

Item	Category	N	%
Sex^a^	Male	15	25.0%
	Female	41	68.3%
Occupation	Health professionals		
	Psychologists^b^	24	40.0%
	Nurses^c^	5	8.3%
	Medical doctors^d^	4	6.7%
	Mental health researchers	4	6.7%
	Occupational therapists	2	3.3%
	Social workers	1	1.7%
	Volunteers^e^	10	23.3%
	Own lived experience	6	10.0%
Age (years)^a^	Range	21–67	
	Mean ± SD	42.9 ± 12.6	
	Median	42.5	

Participants that had professional and personal experience with suicide were assigned to the health professional group

^a^Missing data (*n* = 4)

^b^Includes one psychologist intern

^c^Includes three nurse assistants

^d^Includes medical doctors specialised in children and adolescents

^e^Volunteers from local suicide prevention non-governmental organisations (*Sociedade Amigos da Vida* (*n* = 10; https://sociedadeamigosdavida.org.br/), *Centro de Valorização da Vida* (*n* = 2; https://www.cvv.org.br/), and others (*n* = 2)

### Panel identification and recruitment

The expert panel consisted of health professionals and people with their own lived experience of suicide (also known as consumers) and carers. To be eligible, health professionals and consumers or carers had to be aged 18 years and have had experience with suicide (either personal, professional or as a carer).

A selective sampling method was used to recruit health professionals and consumers. Participants were recruited individually, except for individuals volunteering in a “Centro de Valorização da Vida” helpline (Brazilian suicide prevention non-governmental organisation; NGO), who were encouraged to share the survey link with peers working in the same position. In addition, a wide range of platforms for recruitment were used to increase diversity in our sample, including phone, WhatsApp, and social media groups from various non-governmental organisations focused on suicide prevention and suicide outpatient clinics in the city of Sao Paulo.

### Data collection and analysis

Eligible participants were sent the survey link hosted by Survey Monkey (https://pt.surveymonkey.com/), which could be opened on a computer or a smartphone. The first page of the survey included information about the study. The following pages asked questions about age, sex, current occupation, and type of experience with suicide (professional, personal, or both).

The survey statements used a five-point Likert scale (1 = essential; 2 = important; 3 = depends/don’t know; 4 = not important; 5 = should not be included). Participants were asked to rate how important they believed each statement was for the inclusion in the Brazilian guidelines for someone providing mental health first aid to a person at risk of suicide. Using an open-ended text box at the end of every section, participants were also asked to comment on existing statements or propose new statements. If these were new, and actionable, these were written into items and included in the round 2 survey. In compensation for their time, participants received a small amount of financial compensation (R$100 Brazilian reals; the equivalent of US$20) after completing both rounds of the survey.

After Round 1, statements rated as essential or important by > 80% of participants were immediately included in the guidelines. Conversely, if less than 70% of participants’ statements rated statements as essential or important at round 1, they were immediately excluded from the guidelines. Statements were re-rated in round 2 if they were rated as essential or important by 70–79% of all participants. These cut off values were based on previous studies of culturally adapted MFHA for helping a suicidal person [27–30]. Suggestions made after each section were refined by author AAL, reviewed by authors AVC, TAA, CHMP and added in round 2 for assessment, together with the items for re-rating.

### Guidelines development

Endorsed statements, which were items rated as essential or important by ≥80% of the panellists at round 1, and ≥ 75% at round 2, to ensure sufficient new, culturally appropriate statements were included, were assembled into 10 sections. Duplicate statements or items without novel ideas were deleted. Ambiguous statements were re-worded and refined by two authors (SSR, NR) to improve clarity. The final list of included statements was circulated to the Brazilian team (AAL, SSR) native speakers to refine the wording and generate the final Brazilian guidelines. See the full document of the guidelines in the Additional file 2. 

## Results

### Expert panel information

A total of 60 participants (female 68%) completed the round 1 survey, and 40 of them participated in round 2 (retention rate = 67%). The socio-demographic characteristics of panellists are shown in Table 1. The panellists were aged 21–67 years (mean ± SD = 42.9 ± 12.6, media*n* = 42.5). There were 40 health professionals, including psychologists (*n* = 24), nurses (*n* = 5), medical doctors (*n* = 4), mental health researchers (*n* = 4), occupational therapists (*n* = 2), and one social worker. Another 14 participants were mental health volunteers of Brazilian suicide prevention NGOs, such as Sociedade Amigos da Vida (https://sociedadeamigosdavida.org.br/) and Centro de Valorização da Vida (https://www.cvv.org.br/). A total of six people only had lived experience in suicide; however, several panellists had multiple roles in the field (e.g., medical doctors also working as mental health researchers and psychologists volunteering for mental health NGOs).

### Rating of statements

**Fig. 1:**
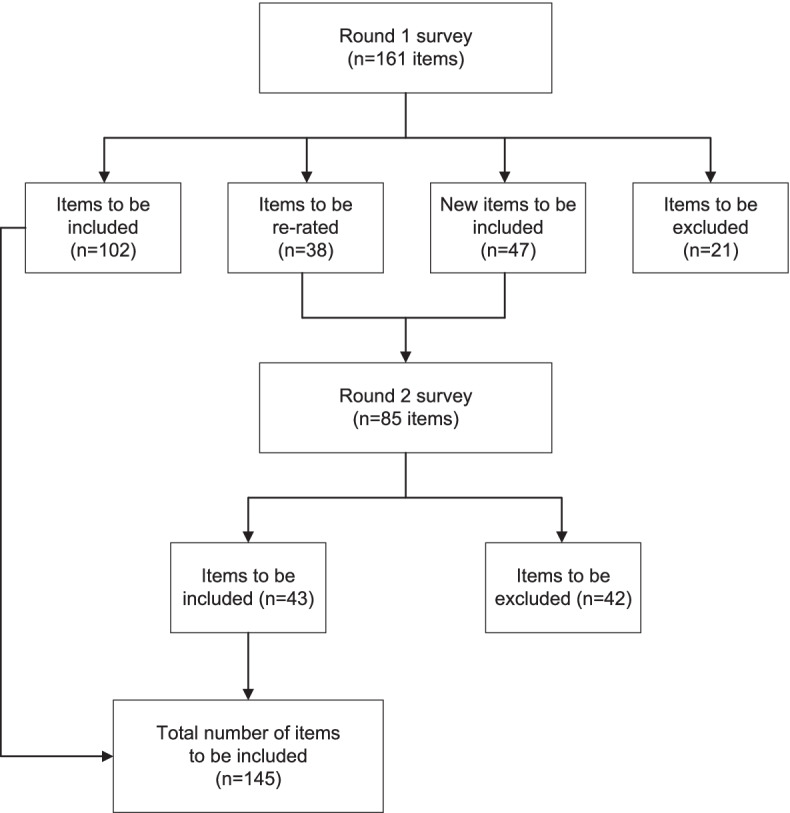
Overview of the study rounds

Consensus was reached on 145 statements for inclusion in the culturally adapted mental health first aid guidelines for Brazil after two survey rounds [see Additional file 1]. The statements were divided into 10 sections in providing mental health first aid to a person at risk of suicide in Brazil: (1) Identification of suicide risk; (2) Assessing seriousness of the suicide risk; (3) Initial assistance; (4) Talking with a suicidal person; (5) No-suicide contracts (safety planning); (6) Ensuring safety; (7) Passing time during the crisis; (8) What the first aider should know; (9) Confidentiality; (10) Adolescent specific items. The number of items included, excluded, re-rated, and endorsed in both rounds is shown in Fig. 1.

A total of 161 items were rated in the round 1 survey, with 102 being endorsed, 38 re-rated, and 21 excluded. Examples of re-rated and excluded items are ‘*The first aider should ask the suicidal person if they have ever known anyone who has died by suicide*’ (endorsement rate = 72%) and ‘*The first aider should ask the suicidal person if they have ever made a suicide plan in the past*’ (endorsement rate = 65%), respectively (an additional Table 1 shows round 1 items and ratings [see Additional file 1]). Re-rated items, combined with another 47 new items created from participants’ comments, were included in round 2. Of these 85 items, 43 items were endorsed and 42 excluded (an additional Table 2 shows round 2 items and ratings (see Additional file 1)). A total of 145 were endorsed to the final guidelines (an additional Table shows the full list of endorsed items [see Additional file 1]).

### Similarities and differences between the English-language and Brazilian guidelines

Beyond including 123 out of 161 items (76%) of the guidelines for English-speaking countries, the Brazilian guidelines included 22 new endorsed items created from the comments of the expert panel. These new endorsed items in the Brazilian guidelines were as follows:

- For ‘Section 1: Identification of suicide risk’ (*n* = 1)
  1. The first aider should try to talk to the person in a quiet and private place.
- For ‘Section 3: Initial assistance’ (*n* = 4)
  2) The first aider should talk to the suicidal person’s family to try to help and follow up the suicidal person, and not let them feel alone.
  3) The first aider should make contact with the family/support network.
  4) The first aider should check the support networks the person has.
  5) The first aider should ask the person what other resources they have to resolve their pain

- For ‘Section 4: Talking with a suicidal person’ (*n* = 7)
  6) The first aider should say they are there to listen without judgement.
  7) The first aider can empathically ‘validate’ the person’s feelings in a suicidal moment, such as “you must be suffering a lot” or “it must be very difficult this moment” making it easier for the suicidal person to talk.
  8) The first aider should have a welcoming listening style and point out the suffering. It is the suffering that needs to disappear, not the life. It is important to understand the person’s suffering.
  9) The first aider should use common terms that can be easily understood by the person.
  10) The first aider should check with the suicidal person if they went through a difficult time, if there was any feeling of rejection and what was the reason they felt rejected.
  11) The first aider should not confront the suicidal person.
  12) The first aider should not mock the person or expose the person to ridicule

- For ‘Section 5: No-suicide contracts (safety planning)’ (*n* = 3)
  13) If possible (and if the suicidal person allows), present and discuss the safety plan with someone close to the suicidal person.
  14) Encourage the person to seek help from someone they trust or from the CVV* (*Centro de Valorização da Vida = Brazilian suicide prevention NGO).
  15) The first aider should check whether the safety plan can be shared with family, friends, or people who the suicidal person trusts that can help.

- For ‘Section 6: Ensuring safety’ (*n* = 1)
  16) The first aider should guide the family to remove objects from the house that could facilitate suicide.

- For ‘Section 7: Passing time during the crisis’ (*n* = 1)
  17) The first aider should ask the person who they enjoy talking to.

- For ‘Section 8: What the first aider should know’ (*n* = 3)
  18) The first aider should be aware that themselves may need psychological assistance after the intervention.
  19) The first aider should know that suicidal people who use drugs become more impulsive.
  20) The first aider needs to know how to deal with the situation so as not to feel guilty if they cannot help the person.

- For ‘Section 9: Confidentiality’ (*n* = 1)
  21) The possibility of a breach of confidentiality in case of risk must be disclosed.

- For ‘Section 10: Adolescent specific items’ (*n* = 1)
  22) Welcome and let the adolescent talk about everything that is concerning them.

Overall, 38 out of 161 (24%) items from English-language guidelines were excluded (round 1 and 2). Most of these items were related to: (1) The first aider using the term suicide, such as in items “*The first aider should ask the suicidal person if they have a plan for suicide*” (rating = 63%) and “*The first aider should demonstrate appropriate language when referring to suicide by using the terms ‘suicide’ or ‘died by suicide*’” (rating = 65%); (2) The first aider discussing with the suicidal person actions they should take to get help (e.g.: “*If the suicidal person is known to have a diagnosis of a mental illness, the first aider should discuss with the suicidal person what actions they should take to get help*” (rating = 62%), “*If the suicidal person does not agree to give the first aider the things they intend using to kill themselves, the first aider should discuss with the suicidal person what actions they should take to get help*” (rating = 58%)); (3) The first aider asking direct questions to the suicidal person, such as “*The first aider should ask the suicidal person if they have decided when they will carry out their plan*” (rating = 60%) and “*The first aider should ask the suicidal person if they have ever known anyone who has died by suicide*” (rating = 57%); (4) The first aider discussing actions for help seeking with the suicidal adolescent, such as “*If the suicidal adolescent can’t commit to stay safe, the first aider should discuss with the suicidal adolescent what actions they should take to get help*” (rating = 68%).

## Discussion

The study aimed to culturally and linguistically adapt the mental health first aid guidelines for helping a person at risk of suicide used in English-speaking countries for Brazil. A two-round Delphi expert consensus method was used and, to our knowledge, this was the first study of its kind conducted in Brazil. While the similarity between the English-language and Brazilian guidelines was modest (76% of English-language statements were endorsed by Brazilian experts), some key differences specific to Brazilian culture were found.

### Explicit use of the term ‘suicide’ and discussing actions for help-seeking

The English-language items using the term ‘suicide’ or discussing with the suicidal person actions for them to get help, such as “*The first aider should demonstrate appropriate language when referring to suicide by using the terms ‘suicide’ or ‘died by suicide*’” and “*If the suicidal person is known to have a diagnosis of a mental illness, the first aider should discuss with the suicidal person what actions they should take to get help*”, were not endorsed in the Brazilian guidelines (ratings were 65 and 62%, respectively). Similar to the recently adapted suicide first aid guidelines for Sri Lanka [28], it is possible that these items received low ratings because of stigmatising attitudes towards suicide and reluctance to openly discuss it [34, 35], as evidence shows that stigma towards mental health problems is present among health professionals in Brazil [13, 14, 36].

Items from the English-language guidelines that involved the first aider asking direct questions to the suicidal person were excluded from the Brazilian guidelines. It is possible that these items were not endorsed as, in the Brazilian culture, there is a tendency not to ask direct questions to avoid conflicts in conversations [37].

Even though some adolescent-specific items from the English-language guidelines were endorsed in the Brazilian guidelines, a few other items were not endorsed, such as “*If the suicidal adolescent can’t commit to stay safe, the first aider should discuss with the suicidal adolescent what actions they should take to get help*” (rating = 68%). It is possible that these items were excluded by panellists as the Latin American culture has a “hierarchy of deference” placing more value and respect on elders than younger people [11, 12], which may be reflected in the lack of discussions with adolescents.

### The role of family and friends

Various new items created from panellists’ comments highlighted the importance of family and friends in the Brazilian adapted guidelines. Examples of these items were “*The first aider should talk to the suicidal person’s family to try to help and follow up the suicidal person, and not let them feel alone*” and “*The first aider should check whether the safety plan can be shared with family, friends, or people who the suicidal person trusts that can help*”. It is possible that these items reflect the idea of familism (a welfare system that places responsibility on the family) in the region [10], as well as the role of the family in the initial stages of help-seeking for mental health difficulties [17]. The importance of friends was also reflected in the adapted guidelines, in line with evidence highlighting the importance of friendship in protecting against suicide in the Brazilian culture [38].

### Self-care of suicide first aiders

The proposed new item “*The first aider should be aware that they themselves may need psychological assistance after the intervention*” and the item from the English-language guidelines “*The first aider must keep in mind that they may not be successful in preventing suicide*” were highly endorsed by panellists (endorsement rates were 95 and 93%, respectively). It is likely that these items were highly endorsed as families may go through “a state of shock” and guilt after a family member suicides [39]. It may also emphasise the importance of postvention for those affected by suicide [40].

### Considerations for future use of the adapted guidelines

This study involved collecting views and opinions from health professionals and individuals with lived experience of suicide in Brazil to adapt the English language guidelines for helping a person at risk of suicide. In doing so, findings showed a modest degree of endorsement of the items in the English-language guidelines, supporting the importance of the guidelines and their adaptation to a different culture. These guidelines may be used to inform MHFA training programs in Brazil. The program may also be useful to reduce stigma in the general population as well as among mental health professionals. It is important, though, to consider further testing and adaptation in the implementation stage of the training within the Brazilian health care system.

### Strengths and limitations

This study had some strengths: firstly, there was a diverse range of health professionals for the expert panel, which ensured diversity in the opinions collected. Secondly, several health professionals also had lived experience of suicide, with one-quarter of the sample being composed of volunteers in suicide prevention organisations. Studies that incorporate the views of people with lived experience are less common in Brazil than in high-income countries and may play a critical role in building capacity in advocacy movements.

A major limitation in this study was that the ratings for the health professionals and the individuals with lived experience were combined in the analysis of Round 1 study data, meaning that information related to rating differences between groups were lost, including information related to the dropout rate from round 1 to round 2.

## Conclusions

A Delphi expert consensus study involving local health professionals and people with lived experience was used to adapt the mental health first aid guidelines for assisting a person at risk of suicide in Brazil. Even though similarities were found to the English-language guidelines, various items related to the explicit use of the term ‘suicide’ and discussing actions for help-seeking were excluded. The adapted guidelines included new items related to family and friends and the self-care of suicide first aiders. Further research on dissemination and uptake of the guidelines in Brazil is necessary as well as research into incorporation of the guidelines into MHFA training for Brazil.

## Supplementary Information

**Additional file 1.**

**Additional file 2.**

## Data Availability

The data supporting our findings is attached as the Additional file 1, which contains all the items that were presented to panellists, including their endorsement rates.

## Abbreviations

LMICs: Low- and middle-income countries
MHFA: Mental Health First Aid
NGOs: Non-governmental organisations
