# Supplementary material for: Cultural adaptation of the mental health first aid guidelines for assisting a person at risk of suicide in Brazil: a Delphi expert consensus study
Source: BMC Psychiatry. 2022 Jun 13;22:397. doi: 10.1186/s12888-022-04042-7 (PMC9195380; doi:10.1186/s12888-022-04042-7)
Supplement: Supplementary file 2 — Additional file 2. [file 12888_2022_4042_MOESM2_ESM.docx]

**DIRETRIZES PARA PENSAMENTOS E COMPORTAMENTOS SUICIDAS NO BRASIL**

O suicídio pode ser prevenido. A maioria das pessoas com ideias suicidas não quer morrer. Elas simplesmente não querem viver com a dor. Falar abertamente sobre pensamentos e sentimentos suicidas pode salvar uma vida. Não subestime suas habilidades para ajudar uma pessoa com ideias suicidas, até mesmo salvar uma vida.

**COMO POSSO SABER SE ALGUÉM ESTÁ TENDO IDEIAS SUICIDA?**

É importante que você conheça os sinais de alerta e os fatores de risco para o suicídio e as razões pelas quais uma pessoa pode ter pensamentos suicidas.

**Sinais de alerta do suicídio:**

- Alguém ameaçando se machucar ou se matar, ou falando em querer morrer
- Alguém procurando maneiras de se matar, como em busca de comprimidos, armas, ou outras formas
- Alguém falando ou escrevendo sobre morte, morrer, ou suicídio
- Desesperança
- Raiva, descontrole, desejo de vingança
- Agindo de forma imprudente ou envolvendo-se em atividades de risco, aparentemente sem pensar
- Sentindo-se preso, como se não houvesse saída
- Aumento do uso de álcool ou drogas
- Afastando-se de amigos, família, e sociedade
- Ansiedade, agitação, incapacidade de dormir ou dormindo o tempo todo
- Mudanças drásticas de humor
- Sem razão pra viver, sem propósito na vida

*Adaptado de*

- *<https://bvsms.saude.gov.br/trabalhando-juntos-para-prevenir-o-suicidio-10-9-dia-mundial-de-prevencao-do-suicidio/>*
- *Rudd et al (2006). Warning signs for suicide: Theory, research and clinical applications. Suicide and Life-Threatening Behavior, 36:255-262*

**FATORES DE RISCO ASSOCIADOS A UM RISCO MAIOR DE SUICÍDIO**

As pessoas correm maior risco de suicídio se tiverem:

- Uma doença mental
- Má saúde física e deficiências
- Tentaram suicídio ou se machucaram no passado
- Coisas ruins aconteceram recentemente, particularmente com relacionamentos ou sua saúde
- Foi abusado fisicamente ou sexualmente quando criança
- Foi exposto recentemente ao suicídio de uma outra pessoa

O suicídio também é mais comum em certos grupos, incluindo homens, indígenas, desempregados, prisioneiros, gays, lésbicas, e bissexuais.

*Adaptado de Hawton K, van Heeringen K. Suicídio. Lancet 2009; 373: 1372-1381.*

**RAZÕES PELAS QUAIS UMA PESSOA PODE TER PENSAMENTOS SUICIDAS**

As principais razões que as pessoas dão para tentar o suicídio são:

1. Necessidade de escapar ou aliviar emoções e pensamentos incontroláveis. A pessoa quer se livrar de uma dor emocional insuportável, sente que sua situação não tem esperança, se sente inútil, e acredita que as outras pessoas estariam melhor sem ela.
2. Desejo de se comunicar ou influenciar um outro indivíduo. A pessoa quer comunicar como se sente a outras pessoas, mudar a forma como as outras pessoas a tratam, ou obter ajuda.

*Adaptado de May & Klonsky (2013). Assessing motivations for suicide attempts: Development of psychometric properties of the inventory of motivations for suicide attempts. Suicide and Life-Threatening Behavior, 43(5), 532-546.*

Se você estiver preocupado que alguém possa estar em risco de suicídio, você precisa abordá-la, e conversar sobre suas preocupações.

**PREPARANDO PARA SE APROXIMAR DA PESSOA**

Esteja ciente de suas atitudes sobre suicídio e do impacto que estas atitudes podem ter sobre sua capacidade de fornecer assistência, como por exemplo: crenças de que o suicídio é errado ou que é uma escolha racional. Saiba que culturas diferentes tem crenças e atitudes sobre o suicídio diferentes das suas.

Esteja ciente de que é mais importante querer genuinamente ajudar do que ter a mesma idade, gênero, ou origem cultural que a pessoa.

**Se você se sentir incapaz de perguntar à pessoa sobre pensamentos suicidas, encontre alguém que seja capaz de fazê-lo.**

**FAZENDO A ABORDAGEM**

Tente conversar com a pessoa num lugar calmo e reservado. Aja prontamente se achar que alguém está considerando suicídio. Mesmo se você tiver apenas um leve suspeita de que a pessoa está tendo pensamentos suicidas, você ainda deve abordá-la.

Conte à pessoa suas preocupações sobre ela, descrevendo os comportamentos da pessoa que fizeram com que você ficasse preocupado sobre o suicídio dela, mas entenda que a pessoa pode não querer falar com você, e neste caso, ofereça ajuda para encontrar alguém com quem ela possa conversar. Caso você se senta incapaz de se conectar com a pessoa, ajude-a a encontrar alguém capaz de fazê-lo.

**PERGUNTANDO SOBRE PENSAMENTOS SUICIDAS**

Qualquer pessoa pode ter pensamentos suicidas. Se você acha que alguém pode estar tendo pensamentos suicidas, você deve perguntar a essa pessoa diretamente. A menos que alguém lhe diga, a única maneira de saber se a pessoa está pensando sobre suicídio é perguntando a ela.

Você pode perguntar, por exemplo:

- “Você está tendo pensamentos suicidas?” ou
- “Você está pensando em se matar?”

Embora seja mais importante fazer a pergunta diretamente do que se preocupar com a exata formulação, você não deve perguntar sobre suicídio de forma de liderança ou julgamento, por exemplo, “Você não está pensando em fazer alguma coisa estúpida, não é?”.

Às vezes as pessoas relutam em perguntar diretamente sobre suicídio porque acham que vão colocar a ideia na cabeça da pessoa. Isso não é verdade. Da mesma forma que se uma pessoa tiver tendo ideias suicidas, perguntar-lhe sobre pensamentos suicidas não aumentará o risco dessa pessoa agir sobre estes pensamentos. Em vez disso, perguntar a pessoa sobre pensamentos suicidas lhe dará a chance de falar sobre seus problemas e mostrar a ela que alguém se importa.

Embora seja comum sentir pânico ou ficar em choque quando alguém manifesta pensamentos suicidas, é importante evitar expressar reações negativas. Faça o seu melhor para parecer calmo(a), confiante, e empático(a) diante da crise suicida, pois isso pode transmitir segurança a pessoa.

**Como devo falar com alguém com ideias suicidas?**

É mais importante ser genuinamente atencioso(a) do que dizer tudo certo. Seja solidário(a) e compreensivo(a) com a pessoa, e ouça plenamente. Os pensamentos suicidas são muitas vezes um pedido de ajuda e uma tentativa desesperada de escapar dos problemas e das angústias.

Pergunte à pessoa o que ela está pensando e sentindo e diga que está ali para escutá-la sem julgar. Assegure a pessoa que você quer ouvir tudo o que ela têm a dizer, que se importa e que está ali para ela, e que quer ajudá-la. Pergunte a pessoa com quem ela gosta de conversar. Permita que a pessoa fale sobre seus pensamentos e sentimentos, e o por que do desejo de querer morrer. Deixe a pessoa saber que não há problema em falar sobre coisas que podem ser dolorosas, mesmo que sejam difíceis. Permita que a pessoa expresse seus sentimentos, por exemplo, permita-a chorar, expressar raiva, ou gritar. A pessoa pode sentir-se aliviada por fazer isto. Incentive a pessoa a falar a maior parte da conversa.

“Valide" o sentimento da pessoa num momento suicida, como, “você deve estar sofrendo muito” ou “deve estar sendo muito difícil este momento” facilitando a pessoa a falar. Tenha um ouvido acolhedor e pontue o sofrimento. O sofrimento precisa desaparecer e não a vida. É importante entender o sofrimento da pessoa.

Lembre-se de agradecer à pessoa por compartilhar com você seus sentimentos e reconheça a coragem que isto requer.

Consulte as caixas abaixo para obter dicas sobre como ouvir de forma eficaz e sobre o que não deve ser feito.

**DICAS DE ESCUTA**

- Seja paciente e calmo(a) enquanto a pessoa fala sobre seus sentimentos
- Dê atenção integral à pessoa
- Use termos que possam ser facilmente compreendidos pela pessoa
- Ouça a pessoa sem expressar julgamento
- Faça perguntas abertas (ou seja, perguntas que não podem ser simplesmente respondidas com “sim” ou “não”) para descobrir mais sobre os pensamentos e sentimentos suicidas e os problemas por trás deles
- Mostre que você está ouvindo o que a pessoa está dizendo resumindo o que foi dito
- Esclareça pontos importantes com a pessoa para garantir que você s se entendam completamente
- Expresse empatia pela pessoa
- Verifique com a pessoa se ela passou por algum momento difícil, se houve algum sentimento de rejeição e por qual motivo se sentia rejeitada
- Preste atenção na sua linguagem corporal, assegurando não passar a impressão de falta de interesse ou de uma atitude negativa
- Seja compreensivo e apoie a pessoa.

**O QUE NÃO FAZER**

**Não…**

- ... brigue ou discuta com a pessoa sobre seus pensamentos suicidas
- ... confronte, ridicularize, ou goze da pessoa
- ... discuta com a pessoa se o suicídio é certo ou errado
- ... use culpa ou ameaças para prevenir o suicídio, por exemplo, não diga à pessoa que ela irá para o inferno ou vai arruinar a vida das pessoas se ela morrer por suicídio
- ... minimize os problemas da pessoa
- ... “tranquilize” a pessoa com comentários simples como “não se preocupe”, “se anime”, “tudo está tão bem na sua vida”, ou “tudo vai ficar bem”
- ... interrompa a pessoa com suas próprias histórias
- ... demonstre falta de interesse ou atitude negativa através de sua linguagem corporal
- ... fale que a pessoa está “blefando”, desafie ou diga que a pessoa “duvido que o faça”
- ... tente dar à pessoa um diagnóstico de problema de saúde mental

Não evite usar a palavra “suicídio”. É importante discutir o assunto diretamente, sem expressar reações negativas a pensamentos suicidas, como atitudes de julgamento, choque, pânico, raiva.

Evite o uso de termos para descrever o suicídio que promovam atitudes estigmatizantes, por exemplo: “cometer suicídio” (implicando que é um crime ou pecado) ou referir-se a uma tentativa de suicídio como tendo “falhado” ou “sem sucesso”, implicando que a morte teria sido um desfecho favorável.

Não deixe que o medo de usar as palavras erradas impeça que voçê incentive a pessoa em questão a falar. Não se deixe ofender por quaisquer ações ou palavras desagradáveis da pessoa.

**COMO POSSO SABER A URGÊNCIA DA SITUAÇÃO?**

Leve toda ideação suicida a sério e tome medidas. Não descarte os pensamentos da pessoa como “para chamar atenção” ou “pedidos de ajuda”. Determine a urgência para agir com base no reconhecimento de sinais de alarme pro suicídio. Pergunte à pessoa sobre questões que afetam sua imediata segurança:

- Se ja está assegurando meios para pôr fim à sua vida
- Se já teve uma tentativa de suicídio no passado

Saiba que a intoxicação pode aumentar o risco da pessoa agir em pensamentos suicidas e pessoas que fazem uso de drogas tornam-se mais impulsivos.

Também é útil descobrir quais apoios estão disponíveis à pessoa:

- Se já contou a alguém como está se sentindo
- Se houveram mudanças de emprego, vida social, ou familiar
- Se recebeu tratamento para problemas de saúde mental ou está tomando algum medicamento.

Esteja ciente de que as pessoas com maior risco de agir em pensamentos de concretizar pensamentos suicidas no futuro próximo são aquelas que têm um plano de suicídio específico, os meios para realizá-lo, um horário determinado para fazê-lo, e a intenção de fazê-lo. No entanto, a falta de um plano de suicídio não é suficiente para garantir a segurança da pessoa.

**COMO POSSO MANTER A PESSOA SEGURA?**

Uma vez que você tenha estabelecido que existe um risco de suicídio, você precisa tomar medidas para manter o pessoa segura. Não deixe sozinha uma pessoa que esteja se sentindo suicida. Trabalhe em colaboração com a pessoa para garantir sua segurança, ao invés de agir sozinho para evitar o suicídio. Converse com a família para tentar ajudar essa pessoa, acompanhá-la e não deixá-la sentir-se sozinha.

Lembre a pessoa que ideias de suicídio não precisam ser colocadas em prática.

Ao falar com a pessoa, concentre-se nas coisas que a manterão segura por enquanto, em vez de as coisas que as colocam em risco. Para ajudar a manter a pessoa segura, desenvolva um plano de segurança com ela (Veja: a caixa abaixo).

**PLANO DE SEGURANÇA**

Um plano de segurança é um acordo entre a pessoa e o socorrista, que envolve ações para manter a pessoa segura. O plano de segurança deve:

- Focar no que a pessoa deve fazer e não no que não deve
- Ser claro, descrevendo o que será feito, quem o fará, e quando será colocado em prática
- Ser por um período de tempo fácil de lidar, de modo que a pessoa sinta-se capaz de cumprir o acordo e ter a sensação de que conseguiu fazê-lo
- Incluir números de contato que a pessoa concorde em ligar, no caso da pessoa sentir-se incapaz de continuar com o acordo, como por exemplo, um profissional da saúde, uma central telefônica de prevenção ao suicídio ou central de crise 24h, amigos e familiares que possam ajudar numa emergência
- Apresentar e discutir o plano de segurança com alguém próximo da pessoa, caso seja possível e a pessoa permita
- Encorajar a pessoa a buscar ajuda com alguém que ela confia ou com alguém do **Centro de Valorização da Vida** (https://www.cvv.org.br/)
- Verificar se o plano de segurança pode ser compartilhado com alguém da família, amigos, ou alguém que a pessoa confie que possa ajudá-la.

Descubra quem ou o que apoiou a pessoa no passado e se esse suporte ainda está disponível. Pergunte a pessoa como ela gostaria de ser apoiada e se há algo que você possa fazer para ajudá-la, mas não tente assumir suas responsabilidades.

Embora você possa oferecer apoio, você não é responsável pelas ações ou comportamentos de uma outra pessoa, e não pode controlar o que ela decidir fazer.

**E ajuda profissional?**

Incentive a pessoa a obter ajuda profissional apropriada o mais rápido possível. Verifique quais redes de apoio que a pessoa tem e quais outros recursos ela/ele tem para sanar a dor. Descubra informações sobre recursos e serviços disponíveis para pessoas em risco de suicídio, como por exemplo, hospitais, clínicas de saúde mental, equipes móveis de atendimento à crise, centrais telefônicas de prevenção ao suicídio, e serviços locais de emergência. Forneça estas informações à pessoa e converse com ela sobre locais onde ela pode buscar ajuda. Se a pessa não quiser falar com ninguém cara a cara, incentive-a a ligar para uma central de prevenção de suicídio ou contate a família/rede de apoio da pessoa.

Não ache que a pessoa irá melhorar sem ajuda ou que ela irá procurar ajuda por conta própria. As pessoas que estão se sentindo suicidas geralmente não pedem ajuda por muitos motivos, incluso estigma, vergonha, crença de que sua situação é desesperadora ou que nada poderá ajuda-lá.

**Se a pessoa estiver relutante em procurar ajuda**, continue incentivando-a a consultar um médico ou entrar em contato com um número de apoio a prevenção ao suicídio para obter orientações de como ajudá-la. **Se a pessoa recusar ajuda profissional**, ligue para um centro de saúde mental ou telefone de emergência e peça conselhos sobre a situação.

Para pessoas em risco mais urgente, ações adicionais podem ser necessárias para facilitar a busca de ajuda profissional. **Se você acredita que a pessoa não ficará segura**, discuta com a pessoa quais os passos que ela deve seguir para conseguir ajuda e/ou peça permissão à pessoa em questão para entrar em contato com o médico ou outro profissional de saúde mental que normalmente trata da pessoa e conte suas preocupações. Se possível, o profissional contatado deve ser um profissional que a pessoa já conheça e confie. **Se a pessoa tiver um plano específico para o suicídio,** ou **se tem meios para realizar o seu plano de suicídio**, ligue para um centro de saúde mental ou telefone de emergência e peça conselhos sobre a situação.

**Se a pessoa já tiver tido uma tentativa de suicídio,** discuta com ela quais os passos que ela deve seguir para conseguir ajuda e a encoraje a procurar ajuda profissional apropriada o mais rápido possível (como, por exemplo, consultar com um profissional de saúde mental ou conversar com alguém num serviço de saúde mental).

**Se a pessoa já tiver um diagnóstico de doença mental**, encoraje a pessoa procurar ajuda profissional apropriada o mais rápido possível (como, por exemplo, consultar com um profissional de saúde mental ou conversar com alguém num serviço de saúde mental).

Certifique-se de não se colocar em perigo ao oferecer apoio à pessoa.

Caso precise chamar a polícia, informe-os que a pessoa está tentando se suicidar para que possam ajudá-lo apropriadamente.

Oriente a família da pessoa a retirar de casa os objetos que possam facilitar o suicídio.

Esteja preparado caso a pessoa expresse raiva e sinta-se traída pela sua tentativa de prevenir o suicídio ou de ajudá-la a obter ajuda profissional. Tente não levar para o lado pessoal nenhuma ação ou palavra da pessoa.

**E SE A PESSOA FOR UM ADOLESCENTE?**

Se o(a) adolescente **não puder se comprometer a se manter seguro(a)** ou tiver **meios de levar adiante** seu plano de suicídio ou já tiver **um diagnóstico conhecido de doença mental:** incentive o(a) adolescente a conseguir ajuda profissional apropriada tão cedo quanto possível, como por exemplo, conversar com um profissional de saúde mental ou alguém em um serviço de saúde mental.

Se o(a) adolescente tiver **pensando em se suicidar** ou tiver um **plano específico** ou estiver **relutante em procurar ajuda**: ligue para um centro de saúde mental ou central telefônica de crise e peça conselhos sobre a situação e/ou incentive o(a) adolescente a conseguir ajuda profissional apropriada tão cedo quanto possível, como por exemplo, conversar com um profissional de saúde mental ou alguém em um serviço de saúde mental.

**Se o(a) adolescente com ideias suicidas tiver tentado suicídio no passado**, discuta com ele(a) quais ações ele(a) deve tomar para conseguir ajuda. **Se o(a) adolescente estiver relutante em procurar ajuda**, continue incentivando-o(a) a conseguir ajuda profissional apropriada.

**Se você não conseguir convencer o(a) adolescente a buscar ajuda**, tente conseguir ajuda de um(a) amigo(a) confiável ou central telefônica de ajuda sobre suicídio ou profissional de saúde mental**. Assegure que o(a) adolescente receba** ajuda de um(a) profissional de saúde, grupo de apoio, ou organização comunitária relevante. **Se o(a) adolescente tiver um plano específico** ou **meios de levar adiante seu plano de suicídio**, discuta com ele(a) quais ações devem ser tomadas para conseguir ajuda.

Acolha e deixe o(a) adolescente falar sobre tudo que o(a) preocupa. Trate o(a) com respeito e envolvê-lo(a) em decisões sobre quem mais sabe sobre a crise de suicídio.

**E SE A PESSOA QUISER QUE EU PROMETA NÃO CONTAR PARA NINGUÉM?**

Você nunca deve concordar em manter um plano de suicídio ou risco de suicídio em segredo. Trate a pessoa com respeito e envolva-a em decisões sobre quem mais sabe sobre a crise suicida. A possibilidade da quebra de sigilo no caso de risco deve ser avisada.

**O QUE DEVO FAZER SE A PESSOA ATUOU EM PENSAMENTOS SUICIDAS?**

Se a pessoa já tiver se ferido, administre os primeiros socorros e ligue para os serviços de emergência, pedindo uma ambulância.

Lembre-se de que, apesar de nossos melhores esforços, podemos não ser bem-sucedidos na prevenção do suicídio. É importante saber lidar com a situação de forma a não se sentir culpado se não conseguir ajudar a pessoa.

**CUIDE-SE**

Depois de ajudar alguém com ideias suicidas, certifique-se de tomar o autocuidado adequado. Fornecendo apoio e assistência a uma pessoa é exaustivo e, portanto, é importante cuidar de você mesmo. Esteja ciente de que você pode precisar de uma assistência psicológica após essa intervenção.

**Uma observação importante:**

A automutilação pode indicar uma série de coisas diferentes. Alguém que está se machucando pode estar em risco de suicídio. Outros se envolvem em um padrão de automutilação ao longo de semanas, meses ou anos e não estão necessariamente com ideias suicidas. Estas diretrizes são para ajudá-lo(a) caso a pessoa que você está ajudando esteja com ideias suicidas. Se a pessoa que você está ajudando está se machucando, mas não tem ideias suicidas, consulte diretrizes em inglês aqui: [*First aid for non-suicidal self-injury*](https://mhfa.com.au/sites/default/files/MHFA_selfinjury_guidelinesA4%202014%20Revised_1.pdf)*.*

**OBJETIVO DESTAS DIRETRIZES**

Estas diretrizes foram elaboradas para ajudar o público a prestar primeiros socorros a alguém que está em risco de suicídio. O papel do socorrista é ajudar a pessoa até que a ajuda profissional seja recebida ou a crise se resolva.

**DESENVOLVIMENTO DESTAS DIRETRIZES**

Estas diretrizes são baseadas nas opiniões de especialistas de pessoas com experiência vivida de psicose (consumidores e cuidadores) e profissionais da saúde mental (clínicos, pesquisadores, e educadores) que são da Austrália, Canadá, Alemanha, Irlanda, Holanda, Nova Zelândia, Suécia, Suíça, Reino Unido, Estados Unidos da América, e Brasil.

**COMO USAR ESTAS DIRETRIZES**

É importante adaptar o seu apoio às necessidades da pessoa que você está ajudando. Esses diretrizes são apenas um conjunto geral de recomendações, e são mais adequadas para fornecer primeiros socorros de saúde mental em países de alta renda com sistemas de saúde desenvolvidos.

Estas diretrizes foram desenvolvidas como parte de um conjunto de diretrizes sobre como ajudar melhor uma pessoa com problemas de saúde mental. Outras diretrizes em inglês podem ser baixadas aqui:

[mhfa.com.au/resources/mental-health-first-aid-guidelines](https://mhfa.com.au/mental-health-first-aid-guidelines).

Embora estas diretrizes sejam protegidas por direitos autorais, elas podem ser reproduzidas livremente para fins sem lucros desde que seja citada. Por favor, cite estas diretrizes da seguinte forma:

Mental Health First Aid Australia. Suicidal thoughts and behaviours: MHFA Guidelines (revised 2014). Melbourne: Mental Health First Aid Australia; 2014.

Dúvidas devem ser enviadas para: [mhfa@mhfa.com.au](mailto:mhfa@mhfa.com.au).
